# Pharmacokinetic Interaction between Pyronaridine-Artesunate and Metoprolol

**DOI:** 10.1128/AAC.02716-14

**Published:** 2014-10

**Authors:** Carrie A. Morris, Rolf Pokorny, Luis Lopez-Lazaro, Robert M. Miller, Sarah Arbe-Barnes, Stephan Duparc, Isabelle Borghini-Fuhrer, Jang-Sik Shin, Lawrence Fleckenstein

**Affiliations:** a College of Pharmacy, University of Iowa, Iowa City, Iowa, USA; b Covance Basel Research Unit AG, Allschwil, Switzerland; c Aptiv Solutions, Stevenage BioScience Catalyst, Stevenage, United Kingdom; d Medicines for Malaria Venture, Geneva, Switzerland; e Shin Poong Pharmaceuticals, Seoul, Republic of Korea

## Abstract

The objectives of this study were to characterize any drug-drug interaction between the antimalarial Pyramax (pyronaridine-artesunate [PA]) and the CYP2D6 probe substrate metoprolol and to assess the safety of 60-day or 90-day PA redosing, particularly with regard to liver biochemistry parameters. Healthy adult subjects were randomized to arm A (*n* = 26) or arm B (*n* = 30), with the arm A subjects administered 100 mg metoprolol tartrate in the first period, 100 mg metoprolol tartrate with the third of three daily doses of PA in the second period, and three daily doses of PA alone in the 90-day redosing period. The arm B subjects received the three-day PA regimen in the first period, with redosing of the regimen after 60 days in the second period. The noncompartmental pharmacokinetic parameters were computed for metoprolol, its metabolite alpha-hydroxymetoprolol, and pyronaridine. The coadministration of metoprolol and PA was associated with an average 47.93% (90% confidence interval [CI], 30.52, 67.66) increase in the maximum concentration of metoprolol and a 25.60% (90% CI, 15.78, 36.25) increase in the metoprolol area under the concentration-time curve from time zero to the last quantifiable concentration obtained (AUC_0-*t*_); these increases most likely resulted from pyronaridine-mediated CYP2D6 inhibition. No interaction effect of metoprolol with pyronaridine was apparent. Following dosing with PA, some subjects experienced rises in liver function tests above the upper limit of normal during the first few days following PA administration. All such elevations resolved typically within 10 days, and up to 30 days at most. In subjects who were redosed, the incidences of alanine aminotransferase (ALT) or aspartate transaminase (AST) level elevations were similar on the first and second administrations, with no marked difference between the 60-day and 90-day redosing.

## INTRODUCTION

Pyramax (pyronaridine-artesunate [PA]) is an artemisinin-based combination therapy indicated for the treatment of acute uncomplicated falciparum and vivax malaria in both children and adults. PA contains the artemisinin derivative artesunate, which effects rapid and profound reductions in parasitemia, and the benzonaphthyridine derivative pyronaridine tetraphosphate, which serves to eliminate residual parasitemia in order to prevent recrudescence, and it helps protect against the loss of artesunate sensitivity (1). Pyronaridine has demonstrated activity against falciparum parasites resistant to other antimalarials, and PA has displayed consistently high efficacy in multiple large clinical trials (2). PA, as a regimen administered once daily for 3 days, received a positive opinion under Article 58 from the European Medicines Agency in February 2012 (1).

The present study was designed to simultaneously address a set of pharmacokinetic and safety objectives regarding PA. The primary pharmacokinetic objective was to characterize any drug-drug interaction between PA and the probe CYP2D6 substrate metoprolol. The concern that such an interaction with CYP2D6 substrates might occur was prompted by human liver microsome findings indicating that pyronaridine is an *in vitro* inhibitor of CYP2D6, with a 50% inhibitory dose (IC_50_) of 1.1 μM. Given that in regions where malaria is highly endemic, malaria reinfections after successful treatment of a prior infection can be frequent, the safety of redosing the PA regimen is an important clinical issue. Therefore, the present study was designed to study the safety of PA redosing after a 60- or 90-day interval, with a particular focus on postdose hepatic aminotransferase level elevations. Such elevations were transient and typically mild when observed during various clinical trials with PA but represent a main toxicity of PA. Given this, the primary safety objective of this study was to assess the effect of 60- or 90-day redosing on elevations of the hepatic biochemistry parameters alanine aminotransferase (ALT) and aspartate aminotransferase (AST).

## MATERIALS AND METHODS

### Subjects.

Healthy male and nonpregnant female volunteers between 18 and 55 years of age, weighing 50 to 90 kg, and with body mass index values between 18.5 and 30.0 kg/m^2^ were considered for inclusion in the study. The included subjects additionally displayed normal ALT, AST, and total bilirubin (TBIL) values at screening, with normal, or abnormal but clinically insignificant, findings allowed for the remaining standard biochemical, hematological, and urine screening laboratory parameters. Subjects meeting any of the following criteria were excluded: known history or evidence of a clinically significant medical disorder, HIV seropositivity, history of tobacco abuse (≥10 cigarettes/day) within the past 2 years, history of hypersensitivity or adverse reaction to pyronaridine, metoprolol, or any artemisinin compound, Gilbert's disease, or known active hepatitis A IgM, hepatitis B surface antigen, or hepatitis C antibody. The included subjects were not to have used systemic medications or herbal products within 14 days of the first study drug administration, although vitamins/minerals could be taken until 4 days predose.

The included subjects were genotyped to determine their CYP2D6 metabolism phenotype; subjects were classified as poor metabolizers (PM), intermediate metabolizers (IM), extensive metabolizers (EM), or ultrarapid metabolizers (URM). All subjects provided written informed consent for their participation in the trial. The study was conducted in accordance with the relevant articles of the Declaration of Helsinki, the International Conference on Harmonisation Good Clinical Practice consolidated guidelines, Swiss Federal Law, and Swiss Regulation on Clinical Trials with Medicinal Products. Prior to the start of the study, the protocol and consent form were reviewed and approved by the EKBB Ethics Committee and Swissmedic.

### Study design and drug administration.

**FIG 1:**
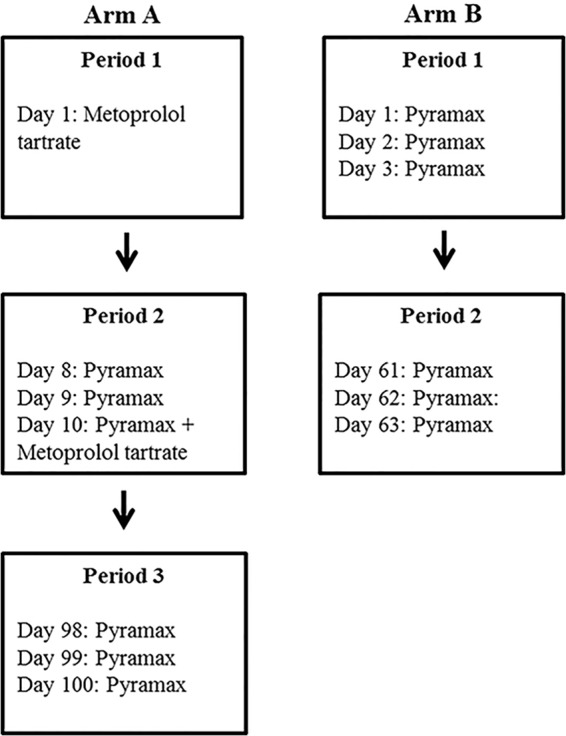
Study drug administration per study design in arms A and B.

The subjects meeting the inclusion criteria were randomized to arm A or arm B of the study. The first two subjects identified as CYP2D6 PM were separately randomized to arms A and B to ensure that at least one PM would be assigned to each study arm. The subjects in arm A participated in the metoprolol interaction and 90-day redosing evaluations, whereas subjects in arm B participated in the 60-day PA redosing evaluation. Figure 1 summarizes the timeline for study drug administration in the two arms.

PA tablets (180 mg pyronaridine tetraphosphate, 60 mg artesunate) and/or 100 mg metoprolol tartrate immediate release tablets, in accordance with the dosing regimen, were swallowed whole with 240 ml of noncarbonated mineral water while the subject was in an upright position. The drug administration occurred following an overnight fast. In both arms, the subjects were administered a PA dose consistent with the label dosing utilized clinically; the dose was administered once daily for three consecutive days. Specifically, subjects weighing <65 kg were administered 3 tablets per day, and subjects weighting ≥65 kg were administered 4 tablets per day.

### Adverse events and clinical laboratory assessments.

The subjects were assessed throughout the study to determine if they were experiencing adverse events. The following clinical laboratory tests related to liver function were performed at multiple points throughout the study: ALT, AST, TBIL, gamma-glutamyl transferase (GGT), alkaline phosphatase (ALP), international normalized ratio (INR), lactate dehydrogenase (LDH), and creatine kinase. In arm A, these tests were performed at screening and on days 1, 2, 7, 9, 10, 11, 12, 13, 15, 22, 29, 36, 43, 50, 97, 99, 100, 101, 102, 103, 105, 112, 119, 126, 133, and 140. In arm B, these tests were performed at screening and on days 1, 2, 3, 4, 5, 6, 8, 15, 22, 29, 36, 43, 60, 62, 63, 64, 65, 66, 68, 75, 82, 89, 96, and 103. When the laboratory test dates coincided with the PA dosing days, blood for the tests was drawn predose.

### Nonredosing criteria.

Subjects were excluded from receiving further study drug, in the context of a given dosing period or of repeat dosing, if they displayed any of the following: ALT or AST levels >3× the upper limit of normal (ULN), TBIL of >2× the ULN, AST or ALT level of >2× the ULN with a TBIL of >1.5× the ULN or INR of >1.4, or AST or ALT level of >2× the ULN with the appearance of fatigue, nausea, vomiting, right upper quadrant pain or tenderness, fever, rash, and/or absolute eosinophilia. The subjects were not given further doses on the second administration if they met the same criteria.

### Pharmacokinetic sampling.

The samples were drawn via an inserted cannula at baseline and for the first 48 h, after which separate venipuncture was employed. Plasma sampling to obtain concentrations of metoprolol and its metabolite by CYP2D6, alpha-hydroxymetoprolol, for noncompartmental analysis was conducted on days 1 (arm A, period 1) and 10 (arm A, period 2). The plasma samples were taken at predose and at 0.25, 0.5, 0.75, 1, 1.5, 2, 3, 4, 6, 8, 10, 12, and 24 h postdose. Blood sampling to obtain pyronaridine concentrations for noncompartmental analysis was conducted on days 10 (period 2) and 100 (period 3) for the subjects in arm A and on days 3 (period 1) and 63 (period 2) for the subjects in arm B. The blood sampling schedule, which was constant across arms and periods, required that a sample be obtained prior to the third PA dose and at 0.5, 1, 1.5, 2, 3, 4, 6, 8, 12, 24, 48, 72, 120, 288, 456, 624, 792, and 960 h following the third dose.

The plasma samples for determining concentrations of artesunate and its metabolite dihydroartemisinin (DHA) were also obtained on days 10 and 100 (arm A) and days 3 and 63 (arm B). These samples were taken prior to the third PA dose and at 1, 3, and 6 h postdose. The intent of this sampling was to screen for atypical artesunate or DHA concentrations. The artesunate and DHA data from a subject having these extreme concentrations were used in a preexisting population pharmacokinetic model to estimate the artesunate and DHA pharmacokinetic parameters of that subject.

### Bioanalytical methods.

Metoprolol and alpha-hydroxymetoprolol sample analyses were performed simultaneously using a liquid chromatography mass spectrometry (LC-MS) assay. The assay was performed with 0.25 ml of plasma, had a quantitation range of 4 to 520 ng/ml for both analytes, and used bisoprolol as an internal standard. Supported liquid-phase extraction with Biotage SLE+ cartridges was used for the sample preparation. Analysis was performed on a Shimadzu LCMS-2010A in single ion monitoring positive mode using atmospheric pressure chemical ionization as the interface. Positive ions were measured using selected ion monitoring mode. The positive ion formed for metoprolol was *m/z* 268.15, and it was *m/z* 284.15 for alpha-hydroxymetoprolol and *m/z* 326.20 for bisoprolol. Chromatography was carried out on a gradient using a Phenomenex Synergi Polar-RP 4-μ 150 by 2.0 mm column with an initial mobile phase of 0.075% formic acid and methanol (75:25) delivered at a flow rate of 0.30 ml/min. The retention times of metoprolol, alpha-hydroxymetoprolol, and bisoprolol were approximately 5.5, 3.2, and 6.2 min, respectively, with a total run time of 8 min.

The metoprolol/alpha-hydroxymetoprolol assay was validated by preparing 3 days of control (12, 100, and 400 ng/ml) and lower limit of quantification (LLOQ) samples in pentuplicate. For both analytes, the intraday and interday bias and variability (% coefficient of variation [%CV]) had magnitudes of <15% for all four assessed concentrations. To assess benchtop stability, control samples were made by spiking plasma at the LLOQ, 100 ng/ml, and 400 ng/ml. The replicates were extracted in pentuplicate at 0, 2, 5, and 24 h. Stability was judged by determining the mean percent difference from the time-zero samples. Metoprolol was stable (i.e., loss <15%) at 24°C for ≥24 h at all levels, and alpha-hydroxymetoprolol was stable at 24°C for ≥5 h at all concentrations.

The recovery of metoprolol was assessed by comparing the area ratio of extracted analyte to unextracted internal standard to the area ratio of unextracted analyte and internal standard, where blank plasma was extracted into the collection tube containing the spiking solutions. The recovery of metoprolol from plasma averaged 76.3%, 73.1%, and 81.6% at 12, 100, and 400 ng/ml, respectively. The recovery of alpha-hydroxymetoprolol for these concentrations averaged 75.7%, 74.9%, and 80.5%, respectively.

Pyronaridine sample analysis was performed using a previously validated LC-MS assay; details regarding the assay, including precision, accuracy, stability, and recovery data, were previously published (3). Briefly, the assay was performed with 0.3 ml whole blood, with amodiaquine used as the internal standard. The quantitation range for the assay of the undiluted samples was 5.7 to 855 ng/ml. Liquid-liquid extraction with ether was used for sample preparation. Analysis was performed on a Shimadzu LCMS 2010A in single ion monitoring mode using atmospheric pressure chemical ionization as the interface. The positive ion for pyronaridine is *m/z* 518.20 and for amodiaquine is *m/z* 356.10. Chromatography was performed using a Gemini 5-μm C_18_ 3.0 by 150 mm column with a 2 mM perfluorooctanoic acid-and-acetonitrile mixture as a mobile phase delivered at a flow rate of 0.5 ml/min. The mobile phase was delivered in gradient mode. The retention times of pyronaridine and amodiaquine are approximately 9.9 and 8.9 min, respectively, with a total run time of 14 min.

The artesunate/DHA sample analysis was performed using a previously validated LC-MS assay slightly modified from a published method (4). Using 0.25 ml of plasma, the quantitation range for the assay of the undiluted samples was 4 to 1,400 ng/ml for artesunate and 2 to 1,400 ng/ml for DHA, with artemisinin used as the internal standard. Solid-phase extraction with Oasis HLB extraction cartridges was used for sample preparation. The analysis was performed on a Shimadzu LCMS-2010A in single ion monitoring positive mode using atmospheric pressure chemical ionization as the interface. Positive ions were measured using selected ion monitoring mode. The positive ion formed for artesunate, alpha-DHA, and beta-DHA was *m/z* 221.05, and for artemisinin, it was *m/z* 283.00. Chromatography was carried on a gradient using a Phenomenex Synergi Max-RP 4-μ 75 by 2.0 mm column with an initial mobile phase of water, methanol, and acetonitrile (40:45:15) delivered at a flow rate of 0.25 ml/min. Methanol (0.3 ml/min) was added postcolumn to improve ionization and prevent probe needle clogging. The retention times of artesunate, alpha-DHA, and artemisinin were approximately 8.0, 5.4, and 6.0 min, respectively, with a total run time of 12 min. The interday and intraday coefficients of variation for artesunate, dihydroartemisinin, and pyronaridine were all <15%.

### Noncompartmental pharmacokinetic analysis methods.

Noncompartmental pharmacokinetic analyses of the metoprolol, alpha-hydroxymetoprolol, and pyronaridine concentrations were conducted using WinNonlin version 5.0 (Pharsight Corporation; Cary, NC, USA). A dose of 105.98 mg was specified when analyzing the alpha-hydroxymetoprolol data in order to account for the increased molecular weight of alpha-hydroxymetoprolol relative to that of metoprolol. The pharmacokinetic parameters for metoprolol and alpha-hydroxymetoprolol were estimated for metoprolol administration without PA (period 1) and with PA (period 2). The pyronaridine pharmacokinetic parameters were estimated utilizing concentrations obtained on the third day of PA administration in periods 2 and 3 (arm A) or periods 1 and 2 (arm B). All calculations used the actual times recorded at the study site. The following parameters were estimated for metoprolol, alpha-hydroxymetoprolol, and pyronaridine: *C*_max_, the peak observed concentration postdose; *T*_max_, time corresponding to the *C*_max_; half-life, computed as ln 2/*k*_el_, with *k*_el_ being the magnitude of the slope of the linear regression of the log concentration versus the time profile during the terminal phase; AUC_0-*t*_, the area under the concentration-time curve from hour 0 through the LQCT, where the LQCT is the time at which the last quantifiable concentration was obtained; for pyronaridine, this represents the area under the curve from administration of the third PA dose through the LQCT; and AUC_0–∞_, AUC from 0 to infinity, computed using the linear trapezoidal rule as AUC_0-*t*_ + *C*_LQCT_/*k*_el_, where *C*_LQCT_ is the last concentration at the LQCT; for pyronaridine, this represents the area under the curve from the administration of the third PA dose through infinity.

The following parameters were estimated for metoprolol and alpha-hydroxymetoprolol only: CL/F, apparent clearance; and *V*_z_/F, apparent volume of distribution.

The following parameters were estimated for pyronaridine only: AUC_0-τ_, the AUC from the time at which the third PA dose was administered to 24 h postdose; and *C*_trough_, the pyronaridine predose concentrations obtained on days 10 and 100 (for arm A) or on days 3 and 63 (for arm B).

For all analytes, the AUC parameters were estimated using a linear-up/log-down method. Furthermore, half-life and AUC_0–∞_ were considered inestimable if the adjusted *R*^2^ associated with regression to obtain the *k*_el_ was <0.85.

### Drug-drug interaction analysis.

For the drug-drug interaction analysis, the pyronaridine (arm A), metoprolol, and alpha-hydroxymetoprolol AUC and *C*_max_ values were logarithmically transformed. For each analyte, a 90% confidence interval (CI) for the difference (second dosing − first dosing) of the transformed values was obtained using the paired *t* test function in SPSS 20 (IBM, Armonk, NY, USA). The results of this analysis were exponentiated and multiplied by 100 to obtain the values included in this report. These results correspond to the 90% confidence intervals for the ratios (second dosing to first dosing) of the geometric means for any given parameter.

For metoprolol, a lack of relevant interaction was specified *a priori* to correspond to the 90% confidence interval for the period 2-to-period 1 ratio for each exposure parameter (AUC_0-*t*_, AUC_0–∞_, and *C*_max_) falling within the range of 80.00 to 125.00%. For pyronaridine, a lack of a relevant interaction was prespecified to correspond to the period 2-to-period 1 90% confidence interval for the exposure parameters (AUC_0-*t*_, AUC_0-τ_, AUC_0–∞_, and *C*_max_) falling within the range of 66.67 to 150.0%.

The more stringent range for metoprolol than for pyronaridine reflects the central intent of the interaction analysis to evaluate any effect associated with the potential of pyronaridine for CYP2D6 inhibition. As metoprolol is simply serving as a probe CYP2D6 substrate in the present study, the 80.00 to 125.00% range is not intended to reflect any judgments regarding clinically relevant differences for metoprolol itself. Furthermore, reasonably stringent limits were desired given the need to define the exposure risk should pyronaridine be given clinically with narrow therapeutic index CYP2D6 substrates. In contrast, the 66.67 to 150.00% range for pyronaridine was constructed to allow for an assessment of the clinically relevant differences in pyronaridine itself. During the clinical development of pyronaridine, doses of 12 mg/kg of body weight have typically been used, with a range of doses in the phase III studies of 6.9 to 13.8 mg/kg. Efficacy appears to decrease at doses of <8 mg/kg; hence, no differences in the effects are expected unless exposure decreases to <⅔ (or 66.67%) of the reference value. The 150.00% value provides the symmetrical upper limit to ⅔ after exponentiation.

### Redosing effect pharmacokinetic analysis.

To evaluate if 60-day and/or 90-day redosing was associated with altered pyronaridine pharmacokinetics, the 90% confidence intervals for the second dosing-to-first dosing ratios of the geometric means were computed for all pyronaridine exposure parameters (AUC_0-*t*_, AUC_0-τ_, AUC_0–∞_, *C*_max_, and *C*_trough_). For arm A, this ratio reflects period 3 to period 2, whereas for arm B, this ratio reflects period 2 to period 1. The methods used to obtain these confidence intervals are analogous to those utilized for the drug-drug interaction analysis above. It should be noted that the arm A ratios for pyronaridine reflected an assessment of both 90-day redosing and the metoprolol coadministration effects on pyronaridine pharmacokinetics.

### CYP2D6 metabolizer status and pyronaridine and metoprolol pharmacokinetics.

To assess for any relationship between pyronaridine pharmacokinetics and CYP2D6 metabolizer status, the plots of pyronaridine pharmacokinetic parameters by metabolizer category were constructed in order to allow for an exploratory analysis of the pharmacokinetic parameters among the metabolizer categories. This process was repeated with the metoprolol results.

### Exploration of relationships between hepatic and pharmacokinetic parameters.

To explore the relationship between the hepatic biochemistry parameters and pyronaridine pharmacokinetics, the peak ALT and AST levels of the patients per dosing period were utilized. These were divided by the appropriate upper limit of normal (ULN) to yield values reflecting a factor times the ULN. For each dosing period, all peak factor × ULN values were plotted against the pyronaridine exposure parameters of the subjects using SPSS 20. These plots were constructed both separately by dosing period and with the data from both dosing periods combined. A visual inspection of these plots was used to explore the possible relationships between ALT or AST levels and the pyronaridine pharmacokinetic parameters.

### Redosing effect hepatic biochemistry evaluations.

The number of subjects in each arm in the initial and redosing intervals displaying elevations in the ALT or AST parameters above the ULN were tabulated as falling between 1× and 3× the ULN, 3× and 5× the ULN, 5× and 10× the ULN, 10× and 20× the ULN, and >20× the ULN. The elevations were also cumulatively tabulated as falling within >1× the ULN, >3× the ULN, and/or 5× the ULN.

### Sample size justification.

A sample size of 22 subjects per arm (assuming 4 losses to follow-up) was considered sufficient to provide an evaluable sample size of 18 subjects. A sample size of 18 subjects was predicted to provide a statistical power of >90% for the study objective of assessing for a drug-drug interaction effect on metoprolol given limits of 80.00 to 125.00%, a with PA-to-without PA ratio of 1, and an estimated intrasubject coefficient of variation of 16.8% for AUC_0–∞_ (and of 15.7% for *C*_max_), as observed by Yuen et al. (5) in their comparison of exposure to a generic and an innovative formulation of metoprolol tartrate in healthy subjects.

The subjects who wished to discontinue the study or withdrew for any reasons other than safety were allowed to be replaced in an effort to ensure that 18 subjects per arm completed the study. The replacement of subjects who withdrew exclusively due to the nonredosing liver biochemistry criteria and who were without associated clinical signs and symptoms was also allowed.

## RESULTS

### Subjects.

Including the replacement subjects, a total of 26 subjects were enrolled in arm A and 30 subjects in arm B. Overall, 12 of the 56 subjects were enrolled into the study as replacements, with 4 such subjects randomized into arm A and 8 into arm B. The timing and reasons for subject discontinuation from the study are summarized in Table 1. The basic summary statistics regarding the subjects included in the pharmacokinetic analyses are given in Table 2.

**TABLE 1:** Reasons for subjects not completing all periods of the study

Time of withdrawal	Reasons for withdrawal in arm:	
	A (*n* = 26)^*a*^	B (*n* = 30)^*b*^
Prior to first PA dosing regimen^*c*^	1 for personal reasons, 1 lost to follow-up	
During first PA dosing regimen	1 for personal reasons, 1 withdrew consent	1 fulfilled nonredosing criteria
Prior to second PA dosing regimen	2 for personal reasons, 2 fulfilled nonredosing criteria	2 for personal reasons, 2 for adverse events (1 unrelated to study drug, 1 related to study drug), 6 fulfilled nonredosing criteria
During second PA dosing regimen	1 for adverse event, 3 fulfilled nonredosing criteria	2 for adverse events, 4 fulfilled nonredosing criteria

a For subjects in arm A, metoprolol was administered on day 1, PA was administered on days 8 to 10, with metoprolol also given on day 10, and PA was administered on days 98 to 100. The number of enrolled patients (*n*) includes replacements.

b For subjects in arm B, PA was administered on days 1 to 3 and again on days 61 to 63. The number of enrolled patients (*n*) includes replacements.

c PA, pyronaridine-artesunate.

**TABLE 2:** Demographic summary statistics for subjects in formal pharmacokinetic statistical analyses

Characteristic^*a*^	Arm A metoprolol analysis	Arm A redosing analysis	Arm B redosing analysis
*n*	22	18	13
Age (yr)	45 ± 7.2	45 ± 8.0	46 ± 8.9
Wt (kg)	74.6 ± 11.4	73.3 ± 11.9	73.5 ± 10.4
Ht (cm)	170.8 ± 8.7	169.6 ± 9.1	171.6 ± 8.1
No. with CYP2D6 metabolization that was:			
Poor	1	1	0
Intermediate	10	8	6
Extensive	10	8	6
Ultrarapid	1	1	1
Gender (no.)			
Male	10	7	7
Female	12	11	6
Ethnicity (no.)			
Asian	1	1	0
Black	1	0	0
Caucasian	12	11	13

a Values are given as mean ± standard deviation unless otherwise specified.

The median dose of pyronaridine was 9.08 mg/kg/day for arm A and 9.19 mg/kg/day for arm B. The doses ranged from 7.96 to 10.03 mg/kg/day and from 8.09 to 10.94 mg/kg/day for arms A and B, respectively. The median doses of artesunate were 3.02 and 3.06 mg/kg/day for arms A and B, respectively, and the doses ranged from 2.65 to 3.34 mg/kg/day in arm A and 2.70 to 3.65 mg/kg/day in arm B. In arm A, all subjects (*n* = 26) received a single oral dose of 100 mg metoprolol (day 1), 24 of 26 subjects received at least a single oral dose of PA in period 2, and of the 24 subjects who received PA, 18 subjects were redosed 90 days later with at least a single oral dose of PA in period 3. In arm B, all subjects (*n* = 30) were exposed to at least a single oral dose of PA in period 1. Of those 30 subjects, 19 were redosed 60 days later with at least a single oral dose of PA in period 2.

### Metoprolol pharmacokinetics.

**FIG 2:**
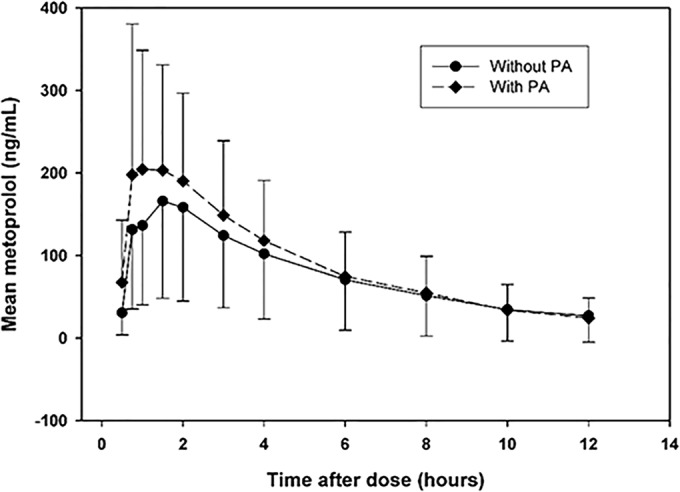
Plot of mean metoprolol concentration versus time after dose. Only time points with ≥50% of concentrations above the LLOQ are plotted. Each error bar represents one standard deviation.

**FIG 3:**
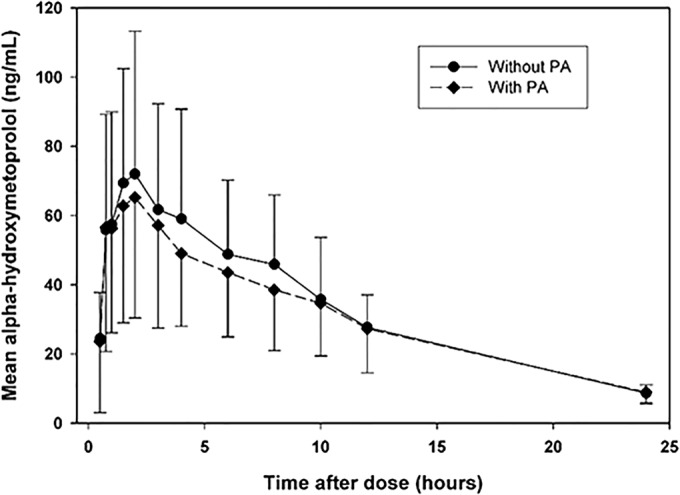
Plot of mean alpha-hydroxymetoprolol concentration versus time after dose. Only time points with ≥50% of concentrations above the LLOQ are plotted. Each error bar represents one standard deviation.

Table 3 presents information regarding the pharmacokinetic parameter estimates obtained for metoprolol and alpha-hydroxymetoprolol during both metoprolol dosing periods. The metoprolol and alpha-hydroxymetoprolol exposure parameters (*C*_max_, AUC_0-t_, and AUC_0–∞_) varied greatly among subjects, even in the absence of PA coadministration; such variability is consistent with the polymorphic nature of CYP2D6 and the consequent effects on metabolism. Considering the geometric means of the parameter estimates, the exposure to metoprolol was increased and the exposure to alpha-hydroxymetoprolol decreased in the presence of PA. This pattern is similarly apparent in Fig. 2 and 3.

**TABLE 3:** Summary statistics for metoprolol and alpha-hydroxymetoprolol pharmacokinetic parameter estimates for subjects completing both periods 1 and 2

Parameter^*a*^	Metoprolol		Alpha-hydroxymetoprolol	
	Without PA (*n* = 22)	With PA (*n* = 22)	Without PA (*n* = 22)^*b*^	With PA (*n* = 22)^*b*^
Half-life (h)	3.43 (27)	3.28 (30)	7.27 (24)	7.63 (30)
*T*_max_ (h)	1.50 (0.75, 3.00)	1.00 (0.75, 2.02)	1.50 (0.75, 8.00)	1.50 (0.42, 6.00)
*C*_max_ (ng/ml)	154.6 (84)	228.6 (62)	69.0 (78)	66.8 (78)
AUC_0-_*_t_* (ng · h/ml)	712 (101)	895 (77)	687 (55)	601 (72)
AUC_0–∞_ (ng · h/ml)	777 (98)	958 (77)	790 (46)	742 (51)
CL/F (liters/kg/h)	1.75 (92)	1.42 (72)	1.80 (52)	1.97 (60)
*V*_z_/F (liters/kg)	8.64 (71)	6.69 (49)	19.7 (83)	21.6 (80)

a For a given parameter, the summary statistics reflect data only from subjects for whom the parameter was estimable in both periods. The values are expressed as the geometric mean (geometric %CV) for all parameters except *T*_max_, which is expressed as the median (range).

b *n* = 19 for alpha-hydroxymetoprolol half-life and AUC_0–∞_.

The 90% confidence intervals for the with PA-to-without PA ratios of the geometric means for all of the metoprolol exposure parameters (*C*_max_, AUC_0-t_, and AUC_0–∞_) fell partly or wholly outside the 80 to 125% range (Table 4). For *C*_max_, the ratio of the geometric means equaled 147.93, with 90% confidence interval limits of 130.52 and 167.66. The effects are less profound for AUC_0-t_ (125.60; 90% CI, 115.78, 136.25) and AUC_0–∞_ (123.32; 90% CI, 114.38, 132.96). However, like *C*_max_, both of these confidence intervals are indicative, per the 80 to 125% criteria, of a drug interaction effect. As indicated in Table 3, the apparent clearance and volume of distribution of metoprolol both decreased, a pattern reflecting the increased oral bioavailability of metoprolol when coadministered with PA, presumably resulting from decreased first-pass metabolism. The geometric mean half-life did not increase with PA administration, as would be anticipated; however, the half-life estimates for metoprolol were based for multiple subjects on the three concentrations occurring over the final 4 h of the sampling period; with this limited time span for concentrations to decline, the resultant half-life estimates are likely not optimally reliable. The 90% confidence intervals for alpha-hydroxymetoprolol parameters appear to display a trend of decreased exposure during the coadministration of metoprolol with PA. For example, the ratio of alpha-hydroxymetoprolol geometric means for the period 2-to-period 1 AUC_0-t_ ratio is 87.46 (90% CI, 79.53, 96.17). As a whole, the findings presented in Tables 3 and 4 are suggestive of PA-mediated CYP2D6 inhibition of metoprolol metabolism.

**TABLE 4:** Ninety percent confidence intervals for the ratios of computed pharmacokinetic parameters of metoprolol and alpha-hydroxymetoprolol obtained following metoprolol administration with PA or without PA

Parameter by drug	*n*	With PA/without PA ratio	Lower limit	Upper limit
Metoprolol				
*C*_max_	22	147.93	130.52	167.66
AUC_0-t_	22	125.60	115.78	136.25
AUC_0–∞_	22	123.32	114.38	132.96
Alpha-hydroxymetoprolol				
*C*_max_	22	96.76	88.57	105.70
AUC_0-t_	22	87.46	79.53	96.17
AUC_0–∞_	19	93.87	85.43	103.15

### PA pharmacokinetics.

**FIG 4:**
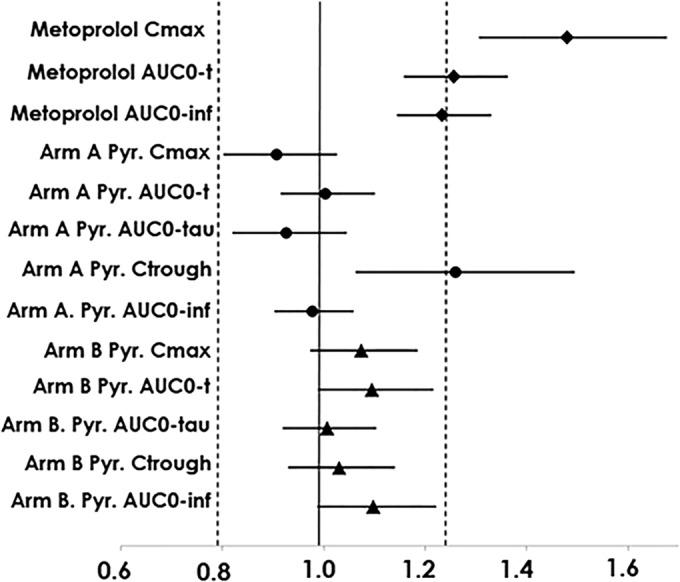
Forest plot of 90% confidence intervals for the ratios of geometric means for pharmacokinetic results. The dashed lines represent the 0.80 and 1.25 boundaries. The solid diamonds indicate point estimates for the geometric mean ratios of with PA to without PA for the metoprolol parameters. The solid circles (arm A) and triangles (arm B) give the point estimates for the geometric mean ratios of second dosing to first dosing for the pyronaridine (Pyr) parameters. The lines extending from the solid bullets indicate the 90% confidence intervals for the ratios of geometric means.

The pyronaridine pharmacokinetic parameter estimates are summarized in Table 5; the geometric mean exposure parameters were highly consistent across arms and periods, with the *C*_max_ geometric means ranging from 330.2 ng/ml (arm B, period 2) to 389.2 ng/ml (arm A, period 2). This consistency is also apparent for the pyronaridine AUC parameter estimates, and it is reflected in the 90% confidence intervals presented in Table 6. As previously noted, the arm A intervals represent an assessment of the effects of metoprolol on pyronaridine pharmacokinetics, as well as 90-day redosing effects. The arm B intervals simply reflect 60-day redosing effects. All of the 90% confidence intervals for pyronaridine fall within the prespecified 66.67 to 150.00% no-relevant-difference range. In actuality, with the exception of the arm A *C*_trough_ interval, a highly variable parameter estimate reflecting single concentration values, all of the 90% confidence intervals for pyronaridine fell within the more stringent 80% to 125% interval employed in the metoprolol analysis. That is, considering the results for the two arms together, and excepting the arm A *C*_trough_, the most extreme lower confidence interval limit belongs to the arm A *C*_max_ (90.70; 90% CI, 80.17, 102.62). The most extreme upper limit belongs to the arm B AUC_0–∞_ (109.76; 90% CI, 98.62, 122.16). A forest plot of these 90% confidence intervals, as well as the corresponding metoprolol results, is provided in Fig. 4. From this plot, the overall lack of any effect of metoprolol or redosing on pyronaridine exposure is apparent. With regard to the artesunate and DHA pharmacokinetics, all of the collected artesunate and DHA samples yielded concentrations consistent with the results from previous noncompartmental and population pharmacokinetics analyses. Therefore, further analysis of these analytes was not pursued.

**TABLE 5:** Summary statistics for pyronaridine pharmacokinetic parameter values obtained in both dosing periods

Parameter^*a*^	Arm A		Arm B	
	First PA dosing (*n* = 14)	Second PA dosing (*n* = 14)	First PA dosing (*n* = 13)	Second PA dosing (*n* = 13)
Half-life (days)	17.3 (44)^*b*^	13.6 (13)^*b*^	14.5 (43)^*c*^	17.5 (34)^*c*^
*T*_max_ (days)	0.062 (0.042, 0.500)	0.062 (0.042, 0.167)	0.062 (0.042, 0.500)	0.062 (0.042, 0.500)
*C*_max_ (ng/ml)	369.5 (23)	335.2 (35)	307.6 (36)	330.2 (32)
AUC_0-t_ (ng · days/ml)	1,078 (20)	1,081 (25)	840 (28)	920 (31)
AUC_0–∞_ (ng · days/ml)	1,272 (16)^*b*^	1,243 (25)^*b*^	988 (27)^*c*^	1,084 (27)^*c*^
AUC_0-τ_(ng · days/ml)	199 (30)	184 (29)	174 (35)	175 (32)
*C*_trough_ (ng/ml)	55.6 (34)^*d*^	70.0 (41)^*d*^	68.4 (41)^*e*^	70.5 (33)^*e*^

a For a given parameter, the summary statistics reflect data only from subjects for whom the parameter was estimable in both periods. The values are expressed as the geometric mean (geometric %CV) for all parameters except *T*_max_, which is expressed as the median (range).

b *n* = 13.

c *n* = 12.

d *n* = 18.

e *n* = 19.

**TABLE 6:** Ninety percent confidence intervals for the ratios of computed pharmacokinetic parameters following redosing compared to initial dosing

Parameter by arm	*n*	Second PA dosing/first PA dosing ratio	Lower limit	Upper limit
Arm A pyronaridine				
*C*_max_	14	90.70	80.17	102.62
AUC_0-t_	14	100.34	91.48	110.05
AUC_0-τ_	14	92.53	81.96	104.46
*C*_trough_	18	125.98	106.24	149.39
AUC_0–∞_	13	97.74	90.24	105.87
Arm B pyronaridine				
*C*_max_	13	107.37	97.31	118.46
AUC_0-_*_t_*	13	109.56	98.75	121.55
AUC_0-τ_	13	100.67	91.85	110.33
*C*_trough_	19	103.01	93.03	114.06
AUC_0–∞_	12	109.76	98.62	122.16

### CYP2D6 phenotype and pharmacokinetics.

**FIG 5:**
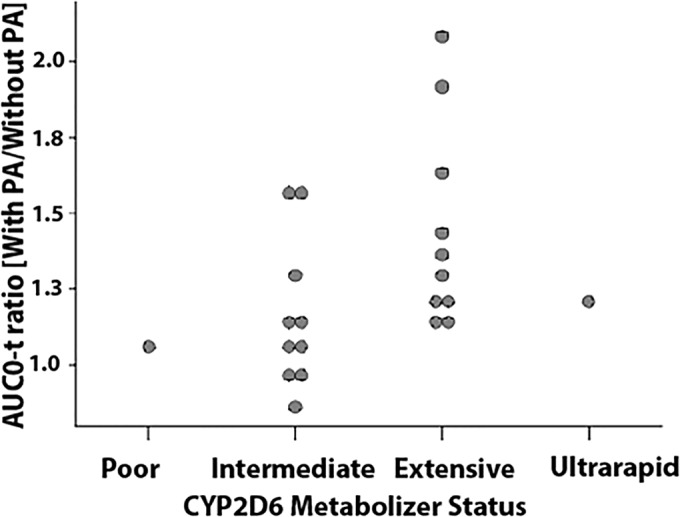
Ratio of metoprolol AUC_0-*t*_ (period 2 to period 1) versus CYP2D6 metabolizer status.

There was no apparent relationship between CYP2D6 phenotype and the pyronaridine exposure parameters (result not shown). However, there was a relationship between the extent of the PA-metoprolol interaction and CYP2D6 phenotype. A plot of the metoprolol AUC_0-t_ with PA-to-without PA ratios is given in Fig. 5; the plots (not shown) for AUC_0–∞_ and *C*_max_ were similar. As indicated by Fig. 5, there was a general trend for the CYP2D6 EM to display a greater increase in metoprolol exposure with PA coadministration than the IM. Given that only one PM and one URM completed the metoprolol dosing, there is insufficient information to draw conclusions regarding the effects of PA coadministration with those phenotypes.

### Exploration of relationship between pyronaridine pharmacokinetic parameters and peak ALT/AST elevations.

Given that the elevations in ALT and AST levels began early in each dosing period (described below), the focus on this analysis was on exploring the possibility of some association between *C*_max_ or AUC_0-τ_ and the peak ALT/AST values, although the plots of all pyronaridine exposure parameters were reviewed as part of this analysis. However, no clear association was detected between the peak ALT or AST values and any pyronaridine estimated exposure parameters (*C*_max_, AUC_0-τ_, AUC_0–∞_, AUC_0-*t*_, and *C*_trough_).

### Individual subject hepatic biochemistry parameters.

Tabulations of the number of subjects who received at least one dose of PA in a given dosing period having elevations from any cause in ALT or AST of >3×, 5×, 10×, or 20× the ULN are provided in Table 7. This table indicates that elevations in ALT and AST levels that are not clinically significant (<3× the ULN) were seen in both arms and both periods; however, elevations in the ALT levels (>3×ULN) that led to the subjects not being redosed occurred in 1 (4%) first administration in arm A (i.e., PA with metoprolol) versus 4 (13%) in arm B (i.e., without metoprolol). For the second administration, during which no subjects were coadministered metoprolol, similar proportions of subjects in each arm displayed ALT level rises of >3× the ULN (17% arm A and 16% arm B), suggesting a lack of difference related to the 60-day versus 90-day redosing intervals. The possibility that metoprolol coadministration is related to this pattern of results is explored below.

**TABLE 7:** Number (%) of subjects in arms A and B displaying elevations in ALT and AST above the ULN due to any cause^*a*^

×ULN by levels	Arm A		Arm B	
	First dosing	Redosing	First dosing	Redosing
*n*	24	18	30	19
ALT				
1–3	10 (42)	5 (28)	11 (37)	5 (26)
3–5		2 (11)	1 (3)	
5–10	1 (4)	1 (6)	3 (10)	2 (11)
10–20				1 (5)
>20			1 (3)	
AST				
1–3	5 (21)	4 (22)	12 (40)	4 (21)
3–5		2 (11)	2 (7)	
5–10	1 (4)	1 (6)		2 (11)
10–20			1 (3)	1 (5)
>20				

a The percentages are based on the number of subjects actually administered at least one dose of PA during a given dosing period.

Overall, for the subjects who were redosed, 43.2% had no increases above the ULN on either administration, 16.2% of the subjects who had an increase after the first administration had no increase after the second, 13.5% who had an increase after the first administration had an increase after the second, and 27% had increases after both administrations.

It is worth noting that none of the subjects with increases in hepatic biochemistry parameters met the criterion defined in the FDA Guidance for premarketing evaluation of drug-induced liver injury for severe liver damage (Hy's law) (6). Notably, none of the subjects with significant increases in the hepatic biochemistry parameters had concomitant values of >1.5× the ULN of the TBIL. In addition, none of the subjects with significant increases in the hepatic biochemistry parameters had values above the ULN for INR. Finally, for all of the subjects with clinically significant elevations in their hepatic biochemistry parameters, the values of those parameters fell below the ULN by the end of the study; typically, the elevations resolved within 10 to at most 30 days.

### Adverse events.

The number of drug-related adverse events reported following metoprolol alone was low, with headache and fatigue being the only two adverse events reported on more than one occasion. The coadministration of metoprolol and PA generally reflected the profile of adverse events observed following PA alone. Overall, there was generally a similar number of adverse events and number of subjects reporting these adverse events from arms A and B. The most frequently reported adverse events following PA were gastrointestinal disorders, elevated liver enzymes (discussed above), and headache. For gastrointestinal disorders, drug-related diarrhea (13 subjects in arm A and 11 subjects in arm B) and nausea (12 subjects in arm A and 11 subjects in arm B) were most frequently reported. Elevated liver enzymes reflect ALT increases (13 subjects in arm A and 16 subjects in arm B) and AST increases (8 subjects in arm A and 17 subjects in arm B). Finally, headache occurred in 11 subjects in arm A and 12 subjects in arm B. There were no adverse events classified as severe or life-threatening. There were two serious adverse events reported, only one of which was considered to be related to the PA study drug, and this was of an exanthematous rash following PA administration on redosing.

In a comparison of the incidence of adverse events during the redosing period and the initial PA dosing period in both arms, there are some apparent differences. For both the arm A and B redosing periods, there was an increase in the number and proportion of subjects reporting the adverse event of vomiting. For arm A, all reported occurrences were in the redosing period, with 4 of the 5 incidences occurring on or prior to 3 days following a reported adverse event of elevated ALT and/or AST levels. For arm B, vomiting adverse events were reported mainly in the redosing period (9 of 12 incidences), where 5 incidences occurred 1 to 3 days prior to elevated ALT and/or AST adverse events, and 1 occurred 3 days prior to elevated GGT levels.

## DISCUSSION

The primary objectives of this study were to assess for a drug-drug interaction between PA and the CYP2D6 probe substrate metoprolol and to assess the safety of 60-day or 90-day redosing, with a particular focus on hepatic biochemistry parameters. The drug interaction analysis provides clear evidence of increased exposure of a CYP2D6 substrate, metoprolol, in the presence of PA. Conversely, the safety analysis presents a more complex set of findings to unravel.

The increased exposure to metoprolol associated with PA coadministration, coupled with the trend toward decreased alpha-hydroxymetoprolol exposure, supports a supposition of pyronaridine-mediated inhibition of CYP2D6. This is consistent with previous *in vitro* analyses suggesting that pyronaridine is a CYP2D6 inhibitor. The average increase in the maximum concentration of metoprolol was 47.93%, with a 25.60% increase for the AUC_0-*t*_. Although such averages align with the *in vitro* finding of CYP2D6 inhibition, their applicability for subjects displaying disparate CYP2D6 phenotypes is somewhat limited. The magnitude of drug-drug interaction effects resulting from CYP2D6 inhibition should theoretically decrease from URM to EM to IM to PM (7). This pattern was somewhat apparent in the metoprolol analysis presented here, with EM overall displaying greater increases in exposure with PA coadministration than that with IM. This differential effect for the various CYP2D6 phenotypes precludes any broad dose adjustment recommendation for CYP2D6 substrates; such substrates include various antiarrhythmics, tricyclic antidepressants, and antipsychotics (7, 8); however, the magnitude of the drug-drug interaction effect observed in the present analysis appears to be sufficiently small, even for subjects with an EM phenotype, to allow for the coadministration of PA with most CYP2D6 substrates under conditions of careful clinical monitoring.

The lack of an effect of metoprolol on pyronaridine pharmacokinetics was anticipated, since metoprolol does not appear to be associated with any clinically relevant metabolizing enzyme inhibition or induction effects (9). Consistent with a lack of a PA redosing effect, the pyronaridine concentrations from the first PA dosing were undetectable for most subjects by the time redosing was initiated both after a 60-day and a 90-day interval. Additionally, given a 15-day half-life for pyronaridine, a 60-day or 90-day interval between the PA dosing regimens provides four or six pyronaridine half-lives, respectively, for pyronaridine elimination. It should be noted that the second dosing-to-first dosing analysis excluded subjects who did not receive or did not complete the second PA dosing due to the occurrence of liver function abnormalities fitting the nonredosing criteria. Whether or not a redosing effect on pyronaridine pharmacokinetics would have been displayed by such subjects cannot be determined from the present data.

Broadly speaking, the safety results do not lend themselves to simple interpretation. Essentially, there is minimal evidence that redosing after either 60 days or 90 days increases the risk of clinically significant ALT or AST level increases. It is challenging to draw strong conclusions given the small number of subjects for analysis, as well as given the elimination of multiple subjects after the first dosing due to fulfillment of the nonredosing criteria. Nonetheless, there are some idiosyncrasies detected in the ALT/AST data that deserve further discussion. The first is the lower rates of hepatic biochemistry parameter elevation among the arm A subjects (who were coadministered metoprolol) than among the arm B subjects during initial dosing. Although the subjects were randomized to the two arms, given the relatively small number of subjects participating in the study, it is possible that, by chance, the subjects more susceptible to the hepatic effects of PA were randomized to arm B. Alternatively, some possible protective effect of metoprolol coadministration can be conjectured. However, such a supposition is somewhat problematic. Specifically, metoprolol was administered only on the third day of PA administration, but hepatic enzyme increases were detected 2 days after the first dose, suggesting that the process leading to ALT/AST level elevations is initiated sometime during the first 2 days of dosing, during which metoprolol was not administered. Furthermore, metoprolol is not known to participate in any pharmacokinetic drug-drug interactions involving CYP2D6, CYP1A2, or CYP3A4, the isozymes likely responsible for metabolizing pyronaridine. That is, metoprolol is not likely to alter the effect of pyronaridine on hepatocytes by acting on the metabolic pathways of pyronaridine. Given these limitations, the possibility that metoprolol was responsible for a protective effect requires further investigation before any conclusions can be drawn.

An overall limitation of this study relates to the extrapolation of the results to the patient population for whom the drug is intended. The incidence of clinically significant hepatic biochemistry elevations is markedly lower in patients administered PA than in healthy subjects. A recent analysis for PA integrated the individual patient profiles from six PA clinical trials, allowing for an evaluation of PA safety across 2,815 patients; the equivalent data from patients administered comparator drugs in the clinical trials were also included. This integrated safety analysis tabulated the rates of liver biochemistry parameter elevations in patients administered PA and the comparator drugs (2). For the patients receiving PA, ALT level elevations of >5× the ULN occurred in only 0.4% (11/2,750) of the patients on day 3 and 0.9% (24/2,709) of the patients on day 7. AST level elevations of >5× the ULN occurred in 0.5% (13/2,757) of the patients on day 3 and 0.3% (13/2,711) of the patients on day 7. These rates were reasonably similar to those of the comparator drugs when used for the treatment of malaria. In the present study, per the data from the combined arms, ALT level elevations of >5× the ULN occurred in 8% of the arm A subjects and 23% of the arm B subjects; AST level elevations of >5× the ULN occurred in 8% and 13% of the arm A and arm B subjects, respectively. The discrepancy between the rates of hepatic biochemistry elevations in healthy and malaria parasitemic subjects may be related to differences in pyronaridine exposure, which is observed to be lower in malaria-infected patients than in healthy subjects (1). That is, this lower exposure to pyronaridine might be linked to the substantially lower rates of significant ALT/AST level elevations in malaria-infected patients.

The findings of the metoprolol analysis, in which PA coadministration increased the metoprolol maximum concentration by 47.93% and the AUC_0-*t*_ by 25.60%, indicate that PA coadministration will likely increase exposure to CYP2D6 substrates. Therefore, caution should be exercised when coadministering PA and CYP2D6 substrates with narrow therapeutic windows. Additionally, the pharmacokinetic analysis suggests that neither metoprolol coadministration nor PA redosing alters pyronaridine pharmacokinetics. The safety analysis suggests that 60-day or 90-day redosing of the PA regimen does not result in a clear increase in the incidence of clinically relevant elevations of the hepatic biochemistry parameters.
